# Noninvasive diagnosis of Ascaris lumbricoides in the common bile duct: A pediatric case report of acute pancreatitis

**DOI:** 10.1002/ccr3.7940

**Published:** 2023-09-22

**Authors:** Komal Samir, Tushar Subash, Arun Subash, Hammad Bilal, Hussain Haider Shah, Tirth Dave

**Affiliations:** ^1^ Dow University of Health Sciences Karachi Pakistan; ^2^ Aga Khan University Karachi Pakistan; ^3^ Baqai medical university Karachi Pakistan; ^4^ Bukovinian State Medical University Chernivtsi Ukraine

**Keywords:** acute pancreatitis, Ascaris lumbricoides, case report, noninvasive approach

## Abstract

**Key Clinical Message:**

This case report highlights the importance of considering parasitic infections, particularly Ascaris lumbricoides, as a possible cause of acute pancreatitis in children, especially in endemic regions. Noninvasive imaging techniques, such as ultrasonography, can play a crucial role in the early detection and diagnosis of this unusual presentation. Timely administration of anthelmintic therapy led to the resolution of symptoms and prevented the need for invasive procedures. Healthcare providers should be vigilant about the diverse clinical manifestations of ascariasis, and regular deworming programs and health education are essential in minimizing the burden of this neglected tropical disease among children.

**Abstract:**

Ascariasis is a common public health problem globally but it is more prevalent in school‐age children and it often goes undiagnosed, leading to severe complications. The purpose of this report is to spread awareness of its unusual presentation and how to judiciously use noninvasive approaches for its diagnosis. We present a case of a 10‐year‐old girl that was presented in pediatric emergency with gradually worsening epigastric pain. Initial lab work‐up showed elevated pancreatic enzymes which lead to the diagnosis of acute pancreatitis. The patient was managed in the line of acute pancreatitis and with further evaluation by imaging techniques such as ultrasound and CT‐scan abdomen, Ascaris lumbricoides (A. lumbricoides) was visualized. She was then treated with prophylactic antibiotics and antiparasitic medications, which resolved her symptoms and the child responded to the treatment. In children, parasites should be considered as a cause of acute pancreatitis by clinicians, especially in low‐income countries, and before performing invasive procedures, noninvasive approaches should be considered as an initial option. This can save the patient from multiple invasive procedure and its severe complications.

## Graphical abstract

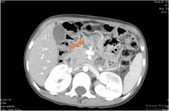

## 1 INTRODUCTION

Acute pancreatitis is a sudden and severe inflammation of the pancreas that presents with a state of severe abdominal pain and vomiting and may lead to a state of multi‐organ dysfunction if not treated on time. Globally, an incidence of 3.6–13.2 cases per 100,000 is reported every year with increased hospital admissions. Most commonly, the causes of acute pancreatitis include gallstones, alcohol intake, steroid use, trauma, autoimmune diseases, or infections in generals.^1^

Ascariasis lumbricoides is the largest helminth‐round worm affecting humans and is one of the leading neglected parasites among children, especially the school‐going age group. It is endemic in developing countries due to poor sanitary conditions^2^ and is common among tropical and subtropical countries. It has affected the population worldwide and in 2021, an estimate of more than 1.5 billion or 24% of the world's population was harbored with ascariasis cases.^3^

A. lumbricoides leads to many acute or chronic manifestations and if left untreated it can be the cause of death among many. Most of the time, it remains asymptomatic and is diagnosed when a child is followed for malnutrition or poor weight gain that affects the child physically as well as academically.^4^ Typically, patients present with either diarrhea or altered bowel habits, worms in vomitus, or signs of intestinal obstruction^5^ but very few extraintestinal presentations have been found. Here we describe a case of unusual presentation of A. lumbricoides being the cause of acute pancreatitis and its noninvasive diagnosis approach.

## 2 CASE REPORT

A 10‐year‐old female child, resident of Landhi, Karachi, presented to the pediatric emergency department (ED) of The Indus Hospital, Karachi at night with a complain of upper abdominal pain for the last 4 days. She was a school‐going child in the 6th grade and used to play in the school playground or later in the evening with her friends daily. Her pain was initially mild to moderate in intensity, colicky in character and intermittent in nature and she had no prior known comorbid. The pain was localized to the epigastric region with no radiation. The patient was able to continue her daily routine but on the day before presenting to the ED, the intensity of pain increased several folds due to which she was unable to go to her school and was given painkillers at home, but her pain did not subside, after which the parents took the child to the Pediatric Emergency Department at The Indus Hospital, Karachi. The pain was not associated with fever, nausea, vomiting, diarrhea, altered bowel habits, or any other symptoms.

In the ED, the child was found to be in severe pain. On physical examination, she appeared pale and poorly nourished. She was vitally stable with a weight of 19 kg, blood pressure of 88/56 mmHg, temperature of 37.5°C, regular pulses with a rate of 98 beats per minute, and a respiratory rate of 26 breaths per minute. Systemic examinations performed included; respiratory examination which revealed equal air entry bilaterally with no added sounds, cardiac examination was unremarkable, there were normal S1 and S2 heart sounds with no other added sounds, murmur, or thrill, central nervous system examination revealed no abnormal neurological signs, and abdominal examination showed severe tenderness and rigidity in the epigastric and left hypogastric region, mild hepatomegaly with smooth borders but no splenomegaly, shifting dullness, or fluid thrill were not appreciable and gut sounds were audible and appeared normal. On admission, a baseline workup was sent, which is presented in Table 1.

**TABLE 1:** Baseline work‐up.

Lab test	Result	Normal range
Hemoglobin	9.0 g/dL	12.0–16.0 g/dL
Red blood cell count	4.68 × 10^12^/L	4.5–5.5 × 10^12^/L
Total leukocyte count	10.15 × 10^9^/L	4.0–11.0 × 10^9^/L
Neutrophils	79%	40%–75%
Lymphocytes	15%	20%–45%
C‐reactive protein	63 mg/L	<5 mg/L
Liver function tests	Within normal range	‐
Creatinine	Within normal range	‐
Serum amylase	274 units/L	28–100 units/L
Serum lipase	351 units/L	10–140 units/L

The patient was shifted to the ward with the impression of acute pancreatitis due to a tender abdomen along with raised serum amylase and lipase levels. Her parents were further inquired about any recent trauma or procedure history, use of any medications, history of mumps, or any recent infection to rule out different causes of acute pancreatitis but nothing significant was report in the history. Following history, a hepatobiliary ultrasonography was performed to rule out any gall stone obstruction, perforation, or congenital pancreatic anomalies. The ultrasound showed hepatomegaly and mildly dilated common bile duct (CBD) with a diameter of 0.8 cm, while the gall bladder, pancreas, kidney, and spleen appeared normal in size and shape.

**FIGURE 1:**
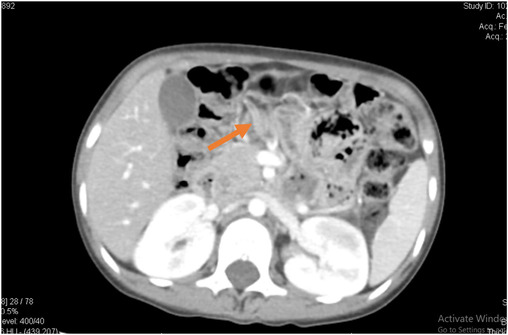
CT abdomen of the patient (axial view).

**FIGURE 2:**
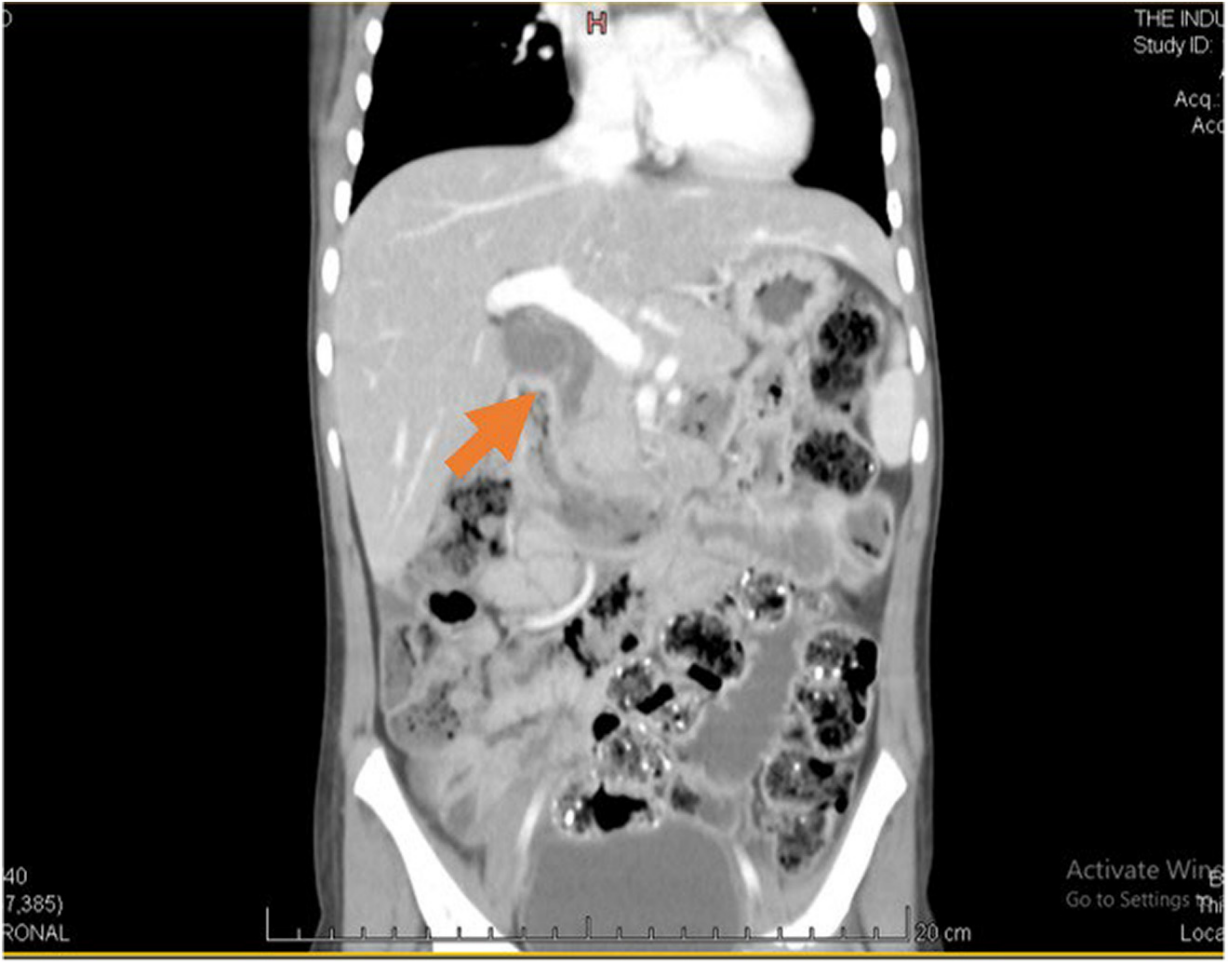
CT abdomen of the patient (coronal view).

Initially, the child was managed on the line of acute pancreatitis, by keeping the child nil per oral and on 100% maintenance fluid. Pain killers were given to relieve the pain, and prophylactic antibiotic injection, ceftriaxone, was started with a dose of 10 mg/kg to treat any possible infectious causes and before starting antibiotics, blood cultures were sent to rule out the causing pathogen. The child's condition did not appear to improve, and her fever started to progressively get more and more severe, while complains of epigastric pain and vomiting continued despite appropriate painkillers and antiemetics. To confirm the cause of the dilated CBD and acute pancreatitis, a Contrast Enhanced Computed Tomography scan (CECT‐scan) of the abdomen was performed the next morning to get a cross‐sectional imaging of the pancreas that is, its anatomical structures and any visible inflammation. CT abdomen with contrast was done under sedation which showed a bulky appearing pancreas with surrounding fat strandings and loss of normal feathery appearance. No evidence of peripancreatic fluid collection or parenchymal necrosis was seen. These findings correlated with mild acute pancreatitis. The CT abdomen also showed the liver to be enlarged with a mildly dilated CBD but no evidence of any calculus within CBD or gall bladder. There were also linear hypodense areas seen in the left proximal jejunum, suggestive of ascariasis (Figures 1 and 2).

**FIGURE 3:**
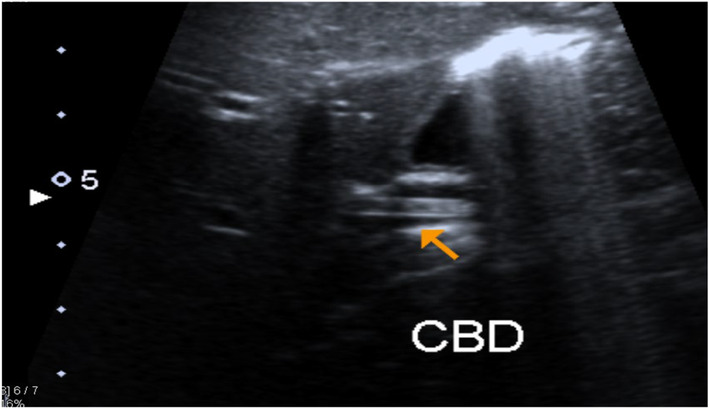
Hepatobiliary ultrasound of the patient.

Upon the suspicion of ascariasis, the decision to deworm the patient was taken in the evening on the same day by using Mebendazole syrup 100 mg twice daily for 3 days, and a pediatric gastroenterologist was consulted for the requirement of any intervention. The gastroenterologist advised to repeat an ultrasound of the hepatobiliary system in the morning and to continue to deworm the patient. Repeat hepatobiliary ultrasound on the next day showed the gall bladder to be normally distended, along with dilated CBD with a diameter of 0.8 cm and evidence of linear tubular structure within CBD extending in the left intrahepatic ducts representing ascariasis (Figure 3). With the deworming continued, the patient's condition started improving and on the third day of admission, the child passed two worms, milky white in color, cylindrical shaped, around 10–12 cm in size, through vomiting, and her symptoms of colicky pain resolved completely afterward. Initially, she was started with clear fluids and later with a fat‐free diet as her lipase and amylase levels were slightly higher than normal but were improved as compared to the initial lab results. She was then sent home as she completed her 3 days dose of mebendazole, she remained symptom‐free for 24 h and started tolerating oral diet. She was given antibiotics as prophylaxis for 5 days during her stay at hospital, until blood cultures reported negative and was called for follow‐up after 10 days in the outpatient pediatrics department with a fresh serum amylase and lipase sample and was advised to take fat‐free diets till her labs get settled.

## 3 DISCUSSION

Ascariasis is the most common helminth parasite around the globe, commonly found in the underdeveloped countries where sanitary conditions and hygiene are least focused on.^2^ The 2016 Global Burden Data reported ascariasis to be the most common soil‐transmitted helminth infection in Pakistan, with a prevalence of 13.1%.^6^ The cases are higher among school‐going children, as they play with soil and in poor hygienic conditions, and not timely deworming of the children, or undertreatment can increase the susceptibility of these cases.^7^ Its mode of transmission is by fecal‐oral route or skin penetration. The fertilized eggs of the species that grow in the intestine invade the mucosal layer and enter systemic circulation.^8^

Ascariasis, a soil‐transmitted helminth infection can present in different ways, depending upon the worm's load. Most of the time, patients remain asymptomatic while sometimes they may present with either acute symptoms of abdominal discomfort or altered bowel habits, or present as a chronic case with symptoms such as cough and dyspnea when the organism involves the pulmonary system, or with malnutrition,^9^ that is, not gaining adequate weight, micronutrient deficiency, and anemia. With increased worm load or chronic cases when worms grow up to adult size, the motile organism can cause multiple complications such as intestinal obstruction^10^ or follow an unusual path and present as acute pancreatitis. It does so by the worm's migration via the ampullary orifice first blocking the pancreatic duct and causing symptoms of pancreatitis and then entering the CBD, where it causes a dilated CBD, as was reported in our patient.^11^

In patients with acute pancreatitis, gallstones, history of trauma, drug use, hypercholesterolemia, and autoimmune diseases are considered some of the common causes among children. When all the above causes rule out, one should think of parasites. Hussain et al. case report demonstrates the worms being the cause of acute pancreatitis in a 25‐year‐old man.^12^ A similar case series has been published in the International Journal of Medical Research & Health Sciences which showed that in cases of acute pancreatitis where other causes are ruled out, endoscopic retrograde cholangiopancreatography (ERCP), an invasive procedure, should be performed, as the investigation to look for ascariasis‐induced acute pancreatitis, but no case has been reported in which ultrasonography, a noninvasive procedure was able to detect the roundworms and was treated empirically.^13^ This should be considered as an initial diagnosis measure, and when suspicion is too high and initial scan is normal, repeat scans should be performed to diagnose and prevent complications of intestinal obstruction to save the patient from invasive procedures and its side effects.

## 4 CONCLUSION

This rare case of Ascaris lumbricoides‐induced acute pancreatitis highlights the importance of considering parasitic infections in the differential diagnosis, especially in endemic regions. Ultrasonography proves to be a valuable noninvasive tool for early detection and guiding empirical treatment, particularly in resource‐constrained settings. Timely administration of anthelmintic therapy resolved symptoms, avoiding unnecessary invasive procedures. Increased awareness among healthcare providers about diverse clinical presentations and the importance of deworming programs can help minimize the burden of helminthic infections. Further studies are needed to explore extraintestinal manifestations and validate ultrasonography as a diagnostic tool. Early detection and appropriate management can significantly improve outcomes for patients with ascariasis‐induced complications.

## AUTHOR CONTRIBUTIONS

**Komal Samir:** Conceptualization; writing – original draft; writing – review and editing. **Tushar Subash:** Writing – original draft; writing – review and editing. **Arun Subash:** Writing – original draft; writing – review and editing. **Hammad Bilal:** Writing – original draft; writing – review and editing. **Hussain Haider Shah:** Writing – original draft; writing – review and editing. **Tirth Dave:** Supervision; writing – original draft; writing – review and editing.

## FUNDING INFORMATION

The author(s) received no financial support for the research, authorship, and/or publication of this article.

## CONFLICT OF INTEREST STATEMENT

The authors declared no potential conflicts of interest with respect to the research, authorship, and/or publication of this article.

## ETHICS STATEMENT

Our institution does not require ethical approval for reporting individual cases or case series.

## CONSENT

Written informed consent was obtained from the patient to publish this report in accordance with the journal's patient consent policy.

## Data Availability

The data that support the findings of this article are available from the corresponding author upon reasonable request.

## References

[1] Restrepo R , Hagerott HE , Kulkarni S , Yasrebi M , Lee EY . Acute pancreatitis in pediatric patients: demographics, etiology, and diagnostic imaging. AJR Am J Roentgenol. 2016;206(3):632‐644. 26901022 10.2214/AJR.14.14223

[2] Galzerano A , Sabatini E , Durì D . Ascaris lumbricoides infection: an unexpected cause of pancreatitis in a western Mediterranean country. East Mediterr Health J. 2010;16(3):350‐351. 20795455

[3] Ali SA , Niaz S , Aguilar‐Marcelino L , et al. Prevalence of Ascaris lumbricoides in contaminated faecal samples of children residing in urban areas of Lahore, Pakistan. Sci Rep. 2020;10(1):21815. 33311542 10.1038/s41598-020-78743-y PMC7733436

[4] Organization WH . Soil‐transmitted helminth infections. 2014 Accessed from: https://www.who.int/news‐room/fact‐sheets/detail/soil‐transmitted‐helminth‐infections

[5] Abdellatif MZ , Belal US , Abdel‐Hafeez EH , Atiya AM , Norose K . Ascaris lumbricoides causing acute abdomen: a case report. East Mediterr Health J. 2013;19(12):1035‐1037. 24684102

[6] Blum AJ , Majid MF , Hotez PJ . Pakistan: a nation held back by NTDs. PLoS Negl Trop Dis. 2018;12(10):e0006751. 30335756 10.1371/journal.pntd.0006751 PMC6193611

[7] Ojja S , Kisaka S , Ediau M , et al. Prevalence, intensity and factors associated with soil‐transmitted helminths infections among preschool‐age children in Hoima district, rural western Uganda. BMC Infect Dis. 2018;18(1):408. 30119650 10.1186/s12879-018-3289-0 PMC6098587

[8] Prevention CfDCa . Ascariasis ‐ Prevention & Control. 2019 Accessed from: https://www.cdc.gov/parasites/ascariasis/prevent.html

[9] Hlaing T . Ascariasis and childhood malnutrition. Parasitology. 1993;107(Suppl):S125‐S136. 8115177 10.1017/s0031182000075557

[10] Mbanga CM , Ombaku KS , Fai KN , Agbor VN . Small bowel obstruction complicating an Ascaris lumbricoides infestation in a 4‐year‐old male: a case report. J Med Case Reports. 2019;13(1):155. 10.1186/s13256-019-2103-y PMC6533677 31122293

[11] Sharma M , Rai P . Pancreatic ascariasis causing acute pancreatitis. Video J Encycl. 2013;1(2):571‐572.

[12] Hussain T , Walizada K , Khan T , Khan R , Mushtaq Z . Ascaris lumbricoides infestation as an unexpected cause of acute pancreatitis. Cureus. 2020;12(12):e12103. 33489520 10.7759/cureus.12103 PMC7805493

[13] Ah R , Gn Y , Fa W , Ra P , Kh C , Ah P . Pancreatitis secondary to Ascaris lumbricoides: a case series analysis. Inte Jour of Medi Res & Health Sci. 2013.

